# On the Applications of EMG Sensors and Signals

**DOI:** 10.3390/s22207966

**Published:** 2022-10-19

**Authors:** Ernest N. Kamavuako

**Affiliations:** 1 Department of Engineering, King’s College London, London WC2R 2LS, UK; ernest.kamavuako@kcl.ac.uk; Tel.: +44-207-848-8666; 2 Faculté de Médecine, Université de Kindu, Kindu, Maniema, Democratic Republic of the Congo

## 1. Introduction

The ability to execute limb motions derives from composite command signals (or efferent signals) that stem from the central nervous system through the highway of the spinal cord and peripheral nerves to the muscles that drive the joints. The brain encodes information about a given movement using electrical impulses, referred to as action potentials. The modulation of commands is possible via the recruitment principle and motor unit firing frequency. Because of direct access to the brain, spinal cord and peripheral nerves pose a significant challenge requiring mainly invasive approaches, and muscles provide excellent access to study motor control. Electromyography (EMG) is a technique used for evaluating and recording electrical activity produced by muscles. EMG signals can be harvested on the skin’s surface, under the skin, and inside the muscle, providing different levels of information. It is regarded as the biological amplifier of nerve impulses, providing an improved signal-to-noise ratio. Over the last few decades, there have been considerable technological advances, including sensor miniaturization and advanced signal processing algorithms. The development has empowered EMG sensors and the associated EMG signals to find applications in many areas. In electrodiagnostic (EDX) medicine, EMG has often been used to cover the entire spectrum of EDX techniques using the needle electrode to record muscle electrical activity. In robotic and rehabilitation applications, the EMG signal captures human intention, creating a communication channel between humans and robots. The EMG is also used to assess swallowing function in motor neuron disorders and estimate fluid intake. Motion analysis, feedback, and handwriting modelling are other important areas which involve the application of EMG sensors and signals. EMG sensors and signals are also used in many research laboratories involved in biomechanics, movement disorders, motor control, postural control, neuromuscular physiology, and physical therapy. This Special Issue attempts to capture the latest advances in EMG sensor development, EMG sensor application, and EMG signal conditioning from theoretical and experimental approaches. Nineteen papers (two reviews and seventeen research papers) have been published, providing useful information on the application of EMG sensors and signals. The 17 research papers addressed several exciting themes: swallowing, motion detection and prosthesis control, muscle synergies, robotic exoskeleton, driver behavior, signal conditioning, and muscle assessment.

## 2. Overview of Contribution

The first review paper [1] presented an in-depth study of 28 articles on TMR-based prosthesis control strategies, consolidating the field’s current knowledge and outlining the limits of these strategies. We learned that there is a lack of accepted reference standard performance evaluation due to several evaluation tests based on different metrics. The diversity of these evaluation tests makes it difficult to define common guidelines used for understanding the potential of the proposed control systems. The second review [2] studied 42 articles. Exactly half of the articles were associated with muscle activity during activities of daily living, and the other half were related to a reduction in the synergy-based dimensionality of EMG. The review identified fundamental challenges and issues relevant to comprehensively understanding human hand behavior, providing more intuitive control of prostheses, and achieving realistic biomechanical models.

Within the swallowing theme, Ye Lin et al. used conditional Granger causality from surface EMG signals to analyze the directed functional coordination between different swallowing muscles in healthy and dysphagia patients swallowing water, saliva, and yoghurt [3]. The study indicated that the analysis of functional coordination supplied relevant information for evaluating motor control synergy and provided a step towards identifying new and robust biomarkers for the early detection of dysphagia. A second paper hinted at the potentialities of surface EMGs to differentiate between liquid and non-liquid swallows and to estimate fluid intake volume [4]. Nevertheless, the performance is dependent on EMG features. Further research is needed to explore this potential, including research in real-life environments. Such a novel system is intended for care homes as a step forward in reducing the rate of dehydration in older adults and improving the quality of care in healthcare settings. Using EMG to monitor diet and fluid intake is viable because there are no privacy issues compared with video- or sound-based monitoring.

Motion detection and prostheses were the topics of seven papers in the Special Issue. A proposed model classifies hand motions and transforms the classification sequence into virtual movement with the Opensim environment [5]. The model was developed using an online database. The model has turned out to be an excellent tool for the practical design of hand prostheses or human-computer based on hand motions. Hagengruber et al. proposed a new labelling approach for proportional EMG-based control that efficiently maps muscular activity to proportional control input with good accuracy [6]. Another study [7] addressed the performance issues caused by electrode shifts, feature vectors, and posture groups. They proposed adding more electrode shift training sessions. Furthermore, the Pearson correlation coefficient helped to select the feature vector. These findings might contribute to the optimization of sEMG-based pattern recognition algorithms.

Commercial prostheses devices use electrodes that can be costly for low-income settings. As a result, low-cost custom-made embroidered EMG electrodes have proven to perform similarly to conventional gel-based EMG electrodes in an online experiment [8], paving the way for low-cost myoelectric sensors. Noor et al. explored the potential use of surface EMG as a controlling mechanism for developing a home-based lower limb rehabilitative device for stroke patients [9]. Experimental data showed a moderate positive correlation between the Fugl–Meyer assessment (FMA) scores and classification accuracies. Using ablation analysis, the authors of [10] investigated the feasibility of using wearable sensors and machine learning to differentiate between standing, walking, running, and sprinting. Results demonstrated that using a reduced sensor set on the lower legs performed similarly to using all sensors. A study effectively summarized the movement detection theme by introducing a system that offers the possibility of longitudinal experiments with advanced prostheses based on a cost-effective Arduino system platform and coupled it with wearable EMG sensors [11]. Home trials with people with limb loss will be carried out in future works.

Muscle synergy was addressed in two studies. One study [12] tested the hypothesis that different subsets of muscle synergies are used in various movement and postural tasks. The approach includes extracting muscle synergies and examining whether these synergies explain each motor task. Their results may support the notion of low dimensionality in motor outputs. The low-dimensionality principle states that the central nervous system flexibly recruits fundamental muscle synergies to execute diverse human behaviors. Another study on muscle synergies [13] proposed a novel muscle synergy extraction method based on multivariate curve resolution–alternating least squares (MCR-ALS). This approach overcomes the limitation of the non-negative matrix factorization (NMF) method used for extracting non-sparse muscle synergy and provides higher repeatability and intra-subject consistency.

Benito de Pedro et al. compare the immediate effectiveness in latent myofascial trigger points between ischemic compression techniques and deep dry needling using surface EMG activity in the lateral and medial gastrocnemius of triathletes [14]. Authors recommended that deep dry needling could be advisable for triathletes who train at speeds lower than 1 m/s. Ischemic compression could be more advisable for training or competitions at speeds greater than 1.5 m/s. Lozano García et al. proposed two noninvasive indices (neuromechanical coupling and mechanical efficiency) of parasternal intercostal muscles [15]. The two indices are used for the regular assessment of patients with disordered respiratory mechanics using noninvasive wearable and wireless devices. The last assessment study [16] presented and validated a framework for continuously assessing fatigue using a system-based monitoring paradigm. Ultimately, monitoring and assessing fatigue has important implications for preventing neuromuscular injury, optimizing training loads, and guiding effective and individualized treatment strategies for rehabilitation.

Robotic exoskeleton driven by EMG has received significant attention in the literature. One study in the Special Issue [17] proposed an artificial neural network-trained adaptive controller mechanism to navigate a wheelchair-mounted upper limb robotic exoskeleton. The EMG from upper limb muscles informs users’ intentions, and thus are the input sources to the system. The authors claimed that the system could be tested on people with muscular dystrophy and neurodegenerative diseases.

One article on driver distraction [18] recommended the use of haptic guidance with adaptive authority for both attentive and distracted drivers because it resulted in a lower driver workload than manual driving and fixed authority. Furthermore, haptic guidance with adaptive authority could reduce lane departure risk.

An interesting paper on statistical analysis [19] proposed a wave train electrical activity method for exploratory data analysis based on 2D and 3D area under curve (AUC) diagrams. The technique was designed to analyze, among many biosignals, EMG data collected from patients with Parkinson’s disease. The aim was to treat the EMG signal as a combination of wave trains and address the generalized characteristics of the EMG signal based on local time–frequency changes in the signal. The proposed method and AUC diagrams could reveal new regularities associated with the high-accuracy diagnosis of Parkinson’s disease.

## 3. Conclusions

This Special Issue demonstrated the breadth of applications to which EMG sensors and signals make extensive contributions. Within the area of swallowing functions, the specific domain of bolus type and fluid volume estimation from EMG has received less attention. Diet and nutrition are key determinants of healthy ageing, within which optimal hydration is important, as a balance between fluids and electrolytes is necessary if cells are to survive and function normally. However, most papers on swallowing functions have aimed to quantify the changes in swallowing biomechanics due to disorders such as dysphagia or stroke. I want to encourage researchers within the field of EMG sensors and applications to contribute to the urgent development of validated methods of assessing fluid intake to support clinical practice and research interventions, and to prevent dehydration, a costly recurrent issue in older adults. The scale of EMG applications provides strong evidence that whenever physiologically appropriate muscles are accessible, EMG sensors should be sought before any other methods, such as access to peripheral nerves.

## Data Availability

Not applicable.
